# Adjuvant Growth Hormone for Ovulation Induction with Gonadotropins in the Treatment of a Woman with Hypopituitarism

**DOI:** 10.1155/2012/356429

**Published:** 2012-07-31

**Authors:** Ariadne Daniel, Shereen Ezzat, Ellen Greenblatt

**Affiliations:** ^1^Department of Reproductive Endocrinology and Infertility, Mount Sinai Center for Fertility and Reproductive Health, 250 Dundas Street West, Suite 700, Toronto, ON, Canada M5T 2Z5; ^2^Division of Endocrine Oncology, University Health Network, 585 University Avenue, Toronto, ON, Canada M5N 2N2

## Abstract

*Objective*. To report the prestimulation use of adjuvant GH for gonadotropin ovulation induction in a woman with hypopituitarism and GH deficiency who previously failed to respond. *Design, Patients, and Measurements*. A 31-year-old nulliparous woman presented with hypopituitarism and GH deficiency after failing ovulation induction with high dose gonadotropins. A trial of GH was undertaken for 5 months prior to ovulation induction resulting in normalization of IGF-I levels. *Results*. Women with hypopituitarism are known to have lower pregnancy rates after ovulation induction with need for higher doses of gonadotropins. A small subset of these patients do not ovulate. This patient had successful ovulation induction and pregnancy with prestimulation GH. *Conclusions*. This case suggests that the use of adjuvant GH in a GH-deficient patient several months before the use of human menopausal gonadotropin results in ovulation and pregnancy.

## 1. Introduction

There is a growing body of literature which suggests that the addition of GH to gonadotropins improves ovarian stimulation and pregnancy rates in poor responders undergoing ovulation induction, and controlled ovarian stimulation for in vitro fertilization [1–3]. Women with hypopituitarism are known to have lower pregnancy rates after ovulation induction with need for higher doses of gonadotropins than with other causes of anovulation [4, 5]. A subset of these women do not respond despite high doses of gonadotropins. In these women, if GH deficiency is discovered, supplementation with GH has been shown to improve chances of ovulation and pregnancy in a few case studies [6]. We report the case of a nulliparous woman with hypopituitarism and GH deficiency who achieved a viable pregnancy with GH priming prior to ovulation induction with hMG after failing high dose hMG alone. 

## 2. Case Report

A 31-year-old Caucasian woman was referred with a history of primary infertility. In 1994, she underwent a transsphenoidal right hemihypophysectomy with a diagnosis of corticotroph tumour. Following surgery, she developed hypopituitarism and secondary amenorrhea. She underwent insulin tolerance testing confirming a GH deficiency and that her cortisol responses had returned to normal. Medical history included class III obesity (BMI 40.3 kg/m^2^), bipolar disorder, and asthma. Her 33-year-old male partner had a normal semen analysis.

She had previously been to another clinic and underwent two cycles of hMG containing a 1 : 1 ratio of urinary FSH : LH, 75 IU of each. The first cycle used a total of 1500 IU and the second cycle used 3750 IU, neither of which resulted in the development of a dominant follicle. A final attempt with hMG was performed in our clinic using a step-up protocol as follows: hMG (Menopur; Ferring, Canada) at 150 IU daily was started on day 3 of an induced menstrual cycle. After 5 days of stimulation her estradiol (E_2_) level was 100 pmol/L and there was no dominant follicle, thus her dose was increased to 225 IU daily. Her dose was increased again 3 days later to 300 IU daily as there was no change in her hormone profile or ultrasound. By day 21 of stimulation, she developed 4 follicles between 1.47–1.64 cm in diameter; however, the E_2_ level was only 329 pmol/L. HCG (Merck, Canada) 10,000 IU was given to stimulate the LH surge and intercourse advised for three nights. Eleven days later her menses started spontaneously. A total of 4125 IU of hMG were used in that cycle.

In light of the massive doses of gonadotropins required to induce ovulation and the low E_2_ per follicle, an antimullerian hormone (AMH) was done to assess her ovarian reserve. The AMH was 37.72 pmol/L, well above the average value expected for her age, demonstrating good ovarian reserve [7, 8].

It was hypothesized that her poor response to gonadotropins may be due to her history of GH deficiency. GH levels were not measured due to its short half-life, instead the more reliable level of IGF-I was used. Her baseline IGF-I level was below the normal range (72 mcg/L; normal 115–307 mcg/L). In consultation with medical endocrinology, a trial of GH (Humatrope; Eli Lilly, Canada) was given for 5 months. The initial dose of Humatrope 0.30 mg subcutaneous daily was increased to 0.50 mg to obtain an IGF-I level in the normal range (253 mcg/L; normal 115–307  mcg/L). Thyroid and adrenal function were monitored with no abnormalities.

Ovulation induction was attempted again using Menopur 375 IU daily started on day 3 of an induced menstrual cycle, and she continued with daily GH. On day 12 of stimulation, 6 follicles measured between 1.40–1.65 cm in diameter and due to a concern with overstimulation, her dose was decreased to Menopur 262.50 IU daily. By day 14 of stimulation, her E_2_ level was 1620 ng/mL and 3 follicles measured 1.40, 1.85, and 1.95 cm in diameter. HCG 10,000 IU was given and an intrauterine insemination performed 36 hours later.

The patient conceived and was seen at 6 weeks and 3 days for an ultrasound revealing one fetal heart rate. Humatrope was stopped at 8 weeks gestation. She has not developed impaired glucose tolerance. Her Cushing's disease remains in remission. The pregnancy is ongoing.

## 3. Discussion

Adjuvant GH has been used in women with hypogonadotropic hypogonadism and GH deficiency to reduce the amount of gonadotropins required for ovulation induction with mixed results [9–11]. There are only a few reports where adjuvant GH has been used in women with GH deficiency resulting in an increased pregnancy rate [12–16]. Most papers describe heterogeneous groups of patients without testing for IGF-I and/or GH levels. 

This case highlights the use of adjuvant GH in an adult GH deficient patient who had previously been resistant to high dose hMG ovulation induction. As with many patients with hypogonadotropic hypogonadism, the response to hMG alone for this patient was poor and did not result in ovulation. This triggered the commencement of GH replacement with confirmation of the normalization of IGF-I levels before ovulation induction. The administration of GH as “priming” before hMG resulted in ovulation and pregnancy. This is congruent with other reports which suggest the importance of prestimulation normalization of IGF-I and/or GH levels [6, 15, 16]. 

With GH treatment there is a reported risk of glucose intolerance and the potential for pituitary/hypothalamic tumour recurrence and cancer. Nevertheless, there have been no reports in the few cases in the infertility literature with short term GH use [17]. Patients on GH with diabetes mellitus must be monitored carefully as changes may need to be made to their antidiabetic medications. Therefore, the use of GH in these women must continue to be investigated. 

While several papers have suggested possible protocols, the most effective dose, start time, and duration of use for GH are not yet standardized in women with hypogonadotropic hypogonadism and GH deficiency. However, the evidence continues to mount for treatment of these women with GH and normalization of IGF-I levels before hMG stimulation for ovulation induction resulting in the highest chance of successful pregnancy.
